# Quantifying social distancing arising from pandemic influenza

**DOI:** 10.1098/rsif.2007.1197

**Published:** 2007-10-04

**Authors:** Peter Caley, David J Philp, Kevin McCracken

**Affiliations:** 1 National Centre for Epidemiology and Population Health, Australian National UniversityCanberra, Australian Capital Territory 0200, Australia; 2 Department of Human Geography, Macquarie UniversitySydney, New South Wales 2109, Australia

**Keywords:** disease reproduction number, *R*_0_, pandemic influenza, social distancing, epidemic attack rate, prior immunity

## Abstract

Local epidemic curves during the 1918–1919 influenza pandemic were often characterized by multiple epidemic waves. Identifying the underlying cause(s) of such waves may help manage future pandemics. We investigate the hypothesis that these waves were caused by people avoiding potentially infectious contacts—a behaviour termed ‘social distancing’. We estimate the effective disease reproduction number and from it infer the maximum degree of social distancing that occurred during the course of the multiple-wave epidemic in Sydney, Australia. We estimate that, on average across the city, people reduced their infectious contact rate by as much as 38%, and that this was sufficient to explain the multiple waves of this epidemic. The basic reproduction number, *R*_0_, was estimated to be in the range of 1.6–2.0 with a preferred estimate of 1.8, in line with other recent estimates for the 1918–1919 influenza pandemic. The data are also consistent with a high proportion (more than 90%) of the population being initially susceptible to clinical infection, and the proportion of infections that were asymptomatic (if this occurs) being no higher than approximately 9%. The observed clinical attack rate of 36.6% was substantially lower than the 59% expected based on the estimated value of *R*_0_, implying that approximately 22% of the population were spared from clinical infection. This reduction in the clinical attack rate translates to an estimated 260 per 100 000 lives having been saved, and suggests that social distancing interventions could play a major role in mitigating the public health impact of future influenza pandemics.

## 1. Introduction

Infectious diseases are commonly controlled by minimizing contact between infectious and susceptible individuals. Personal measures to reduce potentially infectious contacts are sometimes referred to as ‘social distancing’. It has been suggested that policies encouraging social distancing may be effective against pandemic influenza (Bell *et al*. 2006; Glass *et al*. 2006). It is unclear, however, whether individuals can reduce their infectious contact rate to a level low enough to return a worthwhile public health outcome. An examination of levels of social distancing actually achieved during previous epidemics can provide useful guidance as to the effectiveness of social distancing interventions during future influenza pandemics.

The infectiousness of a disease is characterized by the basic reproduction number (*R*_0_), which for our purposes is the expected number of infectious contacts per infective when there are no pharmaceutical or behavioural interventions in place and every individual is equally susceptible. More sophisticated definitions are required where individuals have substantially different risks of infection; the methods described by Diekmann & Heesterbeek (2000) are useful in defining and calculating *R*_0_ when contact structures and other kinds of heterogeneity are important. In practice, when an epidemic occurs, the effective reproduction number (*R*) differs from *R*_0_ due to the deployment of interventions, the build-up of herd immunity and possibly pre-existing immunity.

**Figure 1:**
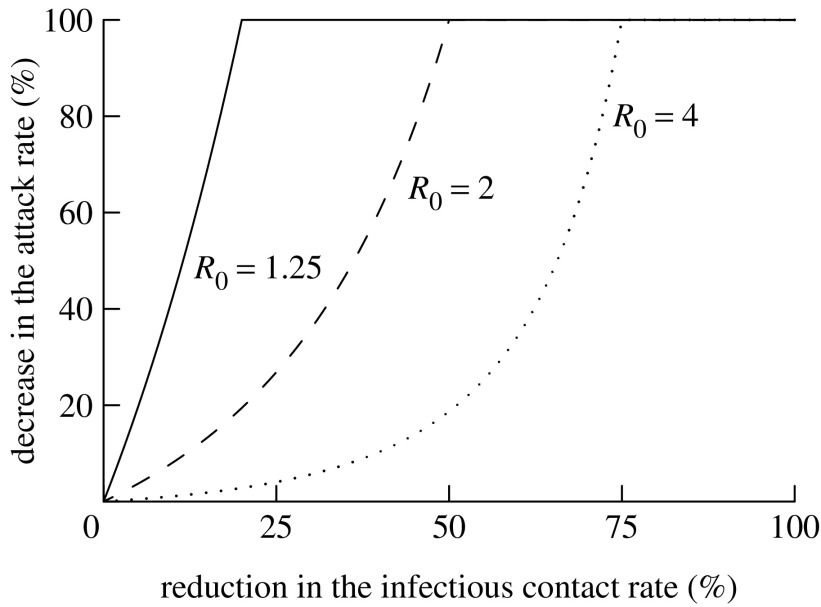
Maximum attainable percentage reduction in the attack rate for epidemics in relation to the extent that the infectious contact rate is reduced across the community. If the intervention is not introduced immediately and sustained indefinitely, a lower reduction will be achieved.

The benefit arising from interventions that additionally decrease *R* beyond that expected based on herd immunity alone may differ depending on the magnitude of the decrease and its timing (Bootsma & Ferguson 2007; Hatchett *et al*. 2007). If a reduction in the infectious contact rate can be introduced early and sustained, the overall attack rate can be reduced. For a given decrease in the contact rate, the relative reduction in the attack rate is smaller for larger *R*_0_ (figure 1). For example, halving the infectious contact rate may lead to a major epidemic being averted (i.e. a 100% reduction in the attack rate) when *R*_0_=2, but, at most, approximately only a 20% reduction in the attack rate if *R*_0_=4 (figure 1).

It is more realistic to assume that interventions to reduce *R* cannot be sustained indefinitely. If interventions are introduced, and subsequently removed before herd immunity has increased sufficiently to reduce *R* to approximately 1, this will postpone and diminish the peak incidence of the epidemic (though not necessarily the eventual attack rate), thus reducing the peak load on health services. Finally, we will argue that if the introduction of time-limited interventions (e.g. social distancing) is timed in such a way as to minimize the number of active infective cases as *R* approaches unity, then the minimum achievable attack rate can be obtained.

Through a combination of geographical isolation and public health measures, the city of Sydney, Australia, delayed the introduction of the Spanish flu by several months until early 1919, at which point public health officials responded almost immediately (McCracken & Curson 2003). As with many populations affected during the 1918–1919 pandemic (e.g. Geneva, Switzerland; Chowell *et al*. 2006), Sydney experienced multiple epidemic waves. There are several theories explaining the multiple waves, including transient post-infection immunity, viral antigenic drift and the involvement of multiple viral strains; substantial counterarguments exist for all these theories and the issue remains unresolved (Taubenberger & Morens 2006). In the case of Sydney, the beginning of a second wave coincided with the lifting of public infection control measures, suggesting that transient adoption of social distancing measures could underlie the observed dynamics (McCracken & Curson 2003). More broadly, Hatchett *et al*. (2007) observed that the quality and timing of non-pharmaceutical public health interventions aimed at decreasing disease transmission by reducing social contact rates appeared to influence the course of influenza epidemics in 17 large US cities during 1918, with second waves occurring only after the relaxation of interventions. We hypothesize that the public of Sydney in 1919 initially responded to the public health measures and subsequently rising and/or high incidence of cases and, particularly, case fatalities by reducing their exposure to potentially infectious contacts. Bootsma & Ferguson (2007) have documented a similar reactive reduction in contact rates in response to high mortality rates arising from pandemic influenza. As the perceived risk decreased, the public subsequently relaxed, returning to normal behaviour. There is a delayed negative feedback between the contact rate and the incidence, and, as with many dynamical systems that experience time lags, oscillations develop. We assume that *R*_0_ is constant over the duration of the epidemic. This is in contrast to Chowell *et al*. (2006) for example, who assumed that *R*_0_ differed between waves—we consider this to be a phenomenological rather than explanatory assumption.

In this paper, we seek to estimate the degree of social distancing that occurred in Sydney in 1919. To do this, we use the epidemic curve and other historical data to estimate (i) the disease reproduction number over the course of the 1919 Sydney influenza epidemic, (ii) bounds on the fraction of people who were asymptomatic seroconverters (whether infectious or not) in that epidemic and (iii) bounds on the fraction of people who were resistant before the epidemic began (e.g. owing to heterotypic immunity).

The methods used in this paper are described in three sections. Section 2 establishes the relevant aspects of the historical background, including why we argue for attributing the epidemic waves to the effect of social distancing. Section 3 measures the reproduction number on each day of the Sydney epidemic by applying the method of Wallinga & Teunis (2004). Section 4 presents methods for using the observed reproduction numbers and the cumulative number of cases to derive relationships between the serological attack rate and the initial fraction of the population that are susceptible. Each of these quantities has direct policy implications for an epidemic. They are often incorporated into models (e.g. Ferguson *et al*. 2005; Longini *et al*. 2005), despite considerable uncertainty about which values are appropriate for pandemic influenza.

## 2. Social distancing, interventions and epidemic waves

In this section, we describe the history of the epidemic in Sydney and what is known about the population's behaviour at each stage. The method we subsequently present in §5 relies on using the historical record to identify periods during the epidemic when the population behaved normally with regard to the transmission of disease. We assume that the public's willingness to reduce transmission relies on their perception of the risk associated with the epidemic.

**Figure 2:**
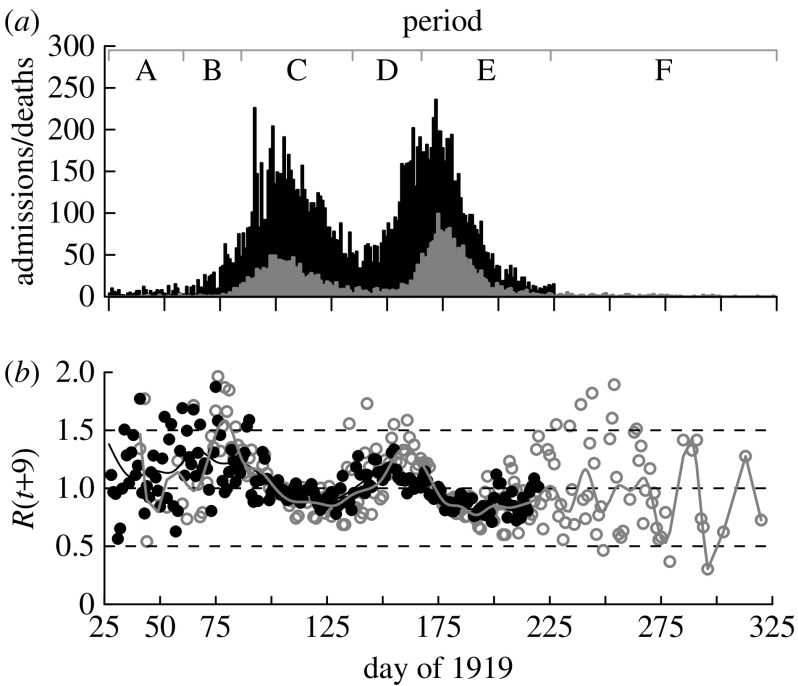
(*a*) Epidemic curve for Sydney 1919 showing daily hospitalizations *h*(*t*) (black bars) and deaths *d*(*t*) (grey bars). Data on hospitalizations were not readily available after day 224. Periods A–F are labelled and characterized as follows: A, first cases, infection control measures; B, threat considered passed, lifting of control measures; C, reimposition of control measures, first wave; D, epidemic considered passed, lifting of control measures; E, second wave; F, epidemic passed. (*b*) Daily effective reproduction numbers estimated from hospitalizations (black circles and black line) and deaths (grey circles and grey line) with a smoothed cubic spline curve. Day *t*=25 is 25 January 1919.

We argue that the historical record, as described by McCracken & Curson (2003), shows periods during which the perceived risk would be high (owing to high infection incidence or the imposition of control measures), and periods when the risk would be perceived as low. Three periods (labelled A, C and E) are associated with a high perceived risk and three others (B, D and F) are associated with a low perceived risk, and consequently normal transmission. Figure 2*a* shows a summary of these periods and a detailed explanation follows.

### 2.1 Public health interventions and social distancing during the epidemic

We define period A as beginning from the time when the first cases were identified (27 January 1919). During this period, extensive infection control measures were imposed, including: closing theatres and public places of entertainment; compulsory wearing of masks on all public transport and in public places; closure of schools; prohibition of race meetings and church services; and removal of patients to hospital and strict quarantine of contact (see McCracken & Curson (2003) for a complete list). As the incidence remained low in comparison with severe epidemics reported from elsewhere around the world, authorities deemed that the threat had passed and most measures were lifted on 1 March.

From 1 March until the reimposition of control measures on 24 March (period B), the incidence rose exponentially. Even so, the daily death rate was low in absolute terms (figure 2*a*) because initial incidence was low, and the mean delay between symptom onset and death was 8.5 days (Armstrong 1920). During this period, we assume that the population approached normal behaviour.

Things changed on the weekend of 22–23 March, when 20 people died of influenza; infection control measures were reimposed around the end of March. We assume that, from 25 March, the perceived severity of the disease was high enough to reduce transmission. These measures were continued throughout the first wave (period C).

We assume that the decreasing incidence led to a decreased perceived risk and that the public started to resume normal behaviour as the authorities lifted infection control measures in the middle of May. We assume that behaviour approached normal during the period D, 25 March to mid-June.

A second wave began shortly after the infection control measures were lifted (i.e. during period D), and was clearly apparent by mid-June. Even though infection control measures were not reimposed, we assume that the high incidence was a sufficient threat to alter people's behaviour. We define this period of altered behaviour (period E) as running from mid-June to 12 August.

We assume that people resumed normal behaviour by 12 August (thereafter period F), as by then the incidence of hospitalizations and deaths had dropped substantially, and the number of hospitalizations ceased to be reported in daily papers.

### 2.2 Why social distancing?

We argue that social distancing is an appropriate explanation for the waves for several reasons. Seasonal changes in virus transmissibility, while possible, cannot be of sufficient size to cause multiple waves—particularly over such a short time period. Indeed, seasonal influenza epidemics on an annual basis cannot occur if the difference attributable to seasons is more than approximately 10% of *R*. Multiple circulating viruses may have contributed to the waves in Europe, where repeat infection was documented (Ministry of Health 1920). However, this could not have occurred in Sydney, where reinfection was extremely rare, and when it did occur the symptoms were mild (Armstrong 1920). Armstrong reports that 814 out of 1488 (55%) health care workers were attacked once, yet only four of these (0.5%) were recorded as being attacked twice.

It might be argued that the first and second waves in Sydney were caused by different strains which provided cross-protection. For this to produce two comparable waves would require that the second strain be substantially more infectious (higher *R*_0_) than the first to overcome the effects of herd immunity. We will show that the reproduction numbers during both waves in Sydney were remarkably similar. Finally, if applied in a transient manner (i.e. applied then lifted too early), there is an underlying mechanistic explanation of resulting waves (Bootsma & Ferguson 2007).

## 3. Estimation of reproduction number

### 3.1 Data collection

Daily hospital admissions attributable to influenza were collated from the *Sydney Morning Herald*, which published a daily report except that data for weekends were not broken into separate days. Daily data on deaths attributable to influenza came from the New South Wales Statistical Register 1919–1920 (table 105). These data have already been given in figure 2. Land and sea border control/quarantine surrounding Sydney meant that the overwhelming majority of cases were not imported.

At the height of the epidemic, the Sydney hospitals were overloaded and turned away patients who would have otherwise been admitted (McCracken & Curson 2003). During the period where the hospitals were not overloaded, the epidemic curve and time-dependent effective reproduction number (see §3.2) can be inferred from either the hospitalization or death data.

### 3.2 Estimation of effective reproduction number *R*

We estimated the effective reproduction number *R*(*t*) for each day of the epidemic using the method of Wallinga & Teunis (2004). The method assumes that the infectiousness function, which describes the rate at which an infected individual transmits infection over the course of their infection (Becker 1989), is known. We derived an average infectivity profile from Ferguson *et al*. (2005) and defined *β*(*a*) to be the average relative infectivity of a person on day *a* of their infection; transmission was assumed not to occur after 10 days. The mean serial interval arising from the resulting infectivity profile was 2.6 days. The method was applied separately to both the death and hospitalization data. Strictly speaking, the method of Wallinga & Teunis (2004) should be applied to incident infections. As infectious events are rarely observed, we (and previous authors) must use symptom onset, death or some other measure as a surrogate marker for infection. Two issues arise, which are as follows. First, notifications of markers (e.g. deaths) may be substantially thinned versions of incident cases. Second, there is a delay, most likely of variable duration, between the infection and the chosen marker. Wallinga & Teunis (2004) showed that a small degree of thinning (e.g. resulting from under-reporting of cases) would not bias estimates of *R*(*t*), but did not investigate the effect of using only a small fraction of cases (as when using deaths as a surrogate when the case-fatality rate is low) to estimate *R*(*t*). In the Sydney 1919 epidemic, the probability of hospitalization and death for a given clinical infection was 4.8 and 1.2%, respectively. We used repeated stochastic simulations of an epidemic with *R*_0_ in the range of 1.5–2.5 in a population of 800 000, with the number of daily cases thinned to 5 and 1% to confirm that thinning *per se* results in no discernible bias in the resulting estimates of *R* over the course of an epidemic.

If the delay from the infection to the chosen marker (e.g. death) is fixed, there is no bias in the resulting estimates of *R*(*t*). Conversely, if there is variability in the delay, then there is a potential for bias, particularly if the distribution of the delay is right-skewed. The effect of the time-to-marker delay distribution is to widen the epidemic curve of the marker, relative to the true incidence curve. The wider the distribution from the infection time to the marker, the greater the potential bias. On theoretical grounds, it is easy to show that during the early and late exponential phases of an epidemic (i.e. its leading or trailing edge), every marker gives an unbiased estimate of *R*, provided that the exponential phase is itself long in duration compared with the width of the distribution for the marked event. During the peaks of the epidemic, the epidemic curve is not exponential and the above result does not apply. In our case, Armstrong (1920) provided data on the distribution of time from the onset of influenza symptoms to death (mean 8.86 days, s.d. 6.0 days), which is well described by a gamma (*k*=2.74, *θ*=3.23) distribution. We repeated our epidemic simulations; this time, modelling the time from infection to death using this gamma distribution shifted 1.5 days to the right to account for the disease incubation period (assumed fixed). Applying the method of Wallinga & Teunis (2004) to the death data confirmed that the resulting daily estimate of *R* has little discernible bias during the early exponential growth period and again during the final days of the epidemic. Our application of the method to death data does underestimate *R* during the middle of the epidemic, and overestimate it at the start of the declining phase; the extent of the bias increases with increasing *R*_0_, though it is less than 10% for a freely evolving epidemic with *R*_0_=1.5. During periods when *R* is close to 1, the bias is also small—this is reflected in the similarity of the hospitalization and death results.

Given that we are predominantly interested in the reproduction numbers during the periods of early exponential growth and during the final cases of the epidemic (§4.5), we consider that the method of Wallinga & Teunis (2004) produces estimates of *R* that are adequate for our purposes. To remove day-to-day variation in estimates of *R*(*t*) for the purpose of making inference, we fitted a smooth curve to the daily estimates of *R*(*t*) using cubic splines with knots every 7 days.

### 3.3 Results for reproduction number *R*

Figure 2*b* shows the daily estimates of the effective reproduction number *R*(*t*) based on both the hospitalization and death data. The estimates are noisier during the periods when case numbers are small (e.g. before day 75 and after day 200). As expected, $\hat{R}(t)$ begins above 1 and drops below 1 as the first wave peaks, though not by much $\left({\hat{R}}_{\text{min}}(C)=0.85\pm 0.01(\pm \text{s}\text{.e}\text{.})\right)$. It returns to greater than 1 at approximately day 130 (figure 2*b*), which is approximately when the second wave of the epidemic began to grow, and remained above 1 until day 165, the peak of the second wave. It is apparent that $\hat{R}(t)$ based on the hospital admissions underestimates *R*(*t*) during both waves due to hospitals being overloaded (figure 2*b*). At times other than early in the epidemic when the number of deaths is very small, the estimates of *R*(*t*) based on either hospitalizations or deaths are very similar (allowing for deaths to lag hospitalized cases; figure 2*b*). We henceforth use deaths only to make inference on *R*(*t*). Indeed, we expect there to be less bias in the estimates of *R*(*t*) arising from deaths compared with hospitalizations. This is because being admitted to hospital is dependent on many factors unrelated to the epidemiology of disease that may vary over time (e.g. perceived need for hospital care based on the case-fatality rate). The maximum value of the smoothed curve during period B gave an estimate of $\hat{R}(\text{B})=1.59\pm 0.02(\pm \text{s}\text{.e}\text{.})$ (figure 2*b*). The mean of the daily reproduction number in period F was $\hat{R}(\text{F})=0.95\pm 0.04(\pm \text{s}\text{.e}\text{.})$.

## 4. Estimation of social distancing and epidemic size

In this section, we present a method for inferring the degree of social distancing during different periods of the epidemic. Our method relies on knowing the reproduction number operating at each time (established in §3). We attribute part of the variation in this reproduction number to herd immunity and the remainder to social distancing.

### 4.1 Available data

The total population size of Sydney was *N*=810 700, of which at least 14 130 (1.74%) were admitted to hospital and approximately 3500 (0.43%) died as a result of influenza infection (McCracken & Curson 2003). Based on a survey of 600 establishments covering 106 923 employees, the proportion of workers that were absent from duty as a result of influenza was 36.6% (Armstrong 1920, p. 144). This was considered as an unbiased estimate of the clinical attack rate, although we argue that the serological attack rate (proportion of workers who developed resistance) may have differed.

### 4.2 Model for *R* in terms of immunity and degree of social distancing

We denote the proportion of the population that were recorded as being hospitalized or as having died on day *t* as *h*(*t*) and *d*(*t*), respectively; these are known from the data. We denote the proportion susceptible as *s*(*t*), and the *per capita* incidence on day *t* as *i*(*t*). We do not assume that infectives were necessarily symptomatic, but they are all assumed to have become immune.

Our model assumes that mixing within the population can be approximated as homogeneous. We assume a form for the effective reproduction number that incorporates the build-up of immunity in the population and social distancing,

(4.1) $$R(t)=R_{0}s(t)\sigma (t),$$

where *σ*(*t*) is a scalar, which describes the extent to which behaviours resulting in disease transmission are maintained. A reduction in *σ*(*t*) indicates that disease transmission has decreased for some reason other than the depletion of susceptibles. For example, when the population is behaving normally (i.e. no social distancing), *σ*(*t*)=1, and when potentially infectious contacts are reduced by half, *σ*(*t*)=0.5. We consider that the population closely approached normal behaviour during periods B and F, and possibly during period D, i.e. *σ*(B)=*σ*(D)=*σ*(F)=1 (table 1).

Our aim is to use this model to estimate *σ*(*t*) by estimating *R*(*t*) and *s*(*t*). More specifically, we seek to estimate

(4.2) $$R_{\text{A}}(t)=R_{0}\sigma (t)=\frac{R(t)}{s(t)},$$

which we refer to as the ‘adjusted reproduction number’—the adjustment referring to the correction of the effective reproduction number for the proportion of the population that are susceptible. When there is no social distancing, $R_{\text{A}}(t)=R_{0}$. Our goal is to estimate how much of the variation in the reproduction number exceeds that which can be attributed to the build-up of immunity, and to attribute that to social distancing. We define *σ*_min_ to be the lowest value of *σ*(*t*) obtained from the analysis, corresponding to the point of greatest social distancing.

### 4.3 Estimation of susceptible fraction *s*(*t*)

The serological attack rate (final proportion infected and developing solid immunity) is *α*=*s*(0)−*s*(∞). The fraction of the population remaining susceptible at time *t* is equal to the initial proportion susceptible−the cumulative proportion infected by *t*,

(4.3) $$s(t)=s(0)-\int_{0}^{t}i(t^{\prime })\text{d}t^{\prime }.$$

**Figure 3:**
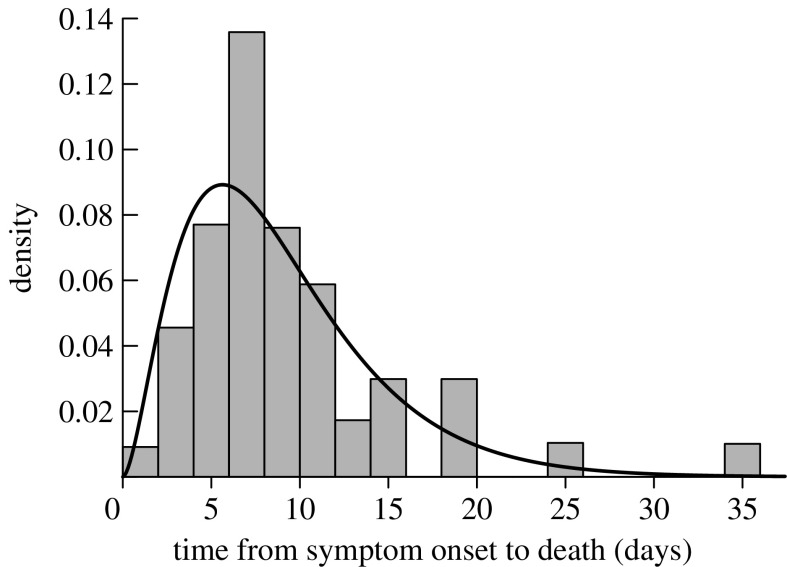
Distribution of time from symptom onset to death for the cases of pandemic influenza in Sydney 1919 (after Armstrong 1920). Fitted curve is a gamma (*k*=2.74, *θ*=3.23) distribution.

We do not observe *i*(*t*) and must infer it from the daily death and/or hospitalization data. In the case of deaths (which in §3.3 we show yields the best estimate of *R*(*t*)), we must account for the time delay (*τ*) between infection and death. The time from symptom onset to death was remarkably similar across all age groups with a mode of 7 days (Armstrong 1920; figure 3). We add 1.5 days for the incubation period (Ferguson *et al*. 2005) and round to the nearest integer, so that *τ*=9. Hence, re-expressing equation (4.3) in terms of daily deaths gives

(4.4) $$s(t)=s(0)-\frac{\alpha }{\pi }\sum_{t^{\prime }=0}^{t^{\prime }+\tau }d(t^{\prime }),$$

where *π* equals the proportion of the population that died as a result of influenza infection. Our main interest in equation (4.4) is to estimate values of *s*(0) and *α* that are compatible with the observed reproduction number over the course of the epidemic.

### 4.4 Effect of social distancing on the attack rate

In this section, we discuss the possible range of values of the serological attack rate. During the preparation of this paper, a similar theory has been presented (Bootsma & Ferguson 2007), which we present in more detail. If social distancing is sufficiently effective (*σ*<1/*R*_0_) and can be maintained, then an epidemic will go extinct by the epidemic threshold theorem (Becker 1989). In a large population, the fraction who become infected in this case is negligible. This may have contributed to the extinction of SARS virus (Riley *et al*. 2003).

If an epidemic cannot be contained by social distancing, and goes on to infect a sizeable fraction, the serological attack rate *α* must lie between a minimum value *α*_min_ and a maximum value *α*_max_. Consider two hypothetical major epidemics, the first without social distancing and the second with what we will argue is optimum effective social distancing. For an epidemic in a reasonably well-mixed population, unimpeded by social distancing, *α*_max_ is obtained from *R*_0_ and *s*(0) by the final size equation (Diekmann & Heesterbeek 2000)

(4.5) $$\alpha_{\text{max}}=s(0)(1-{\text{e}}^{-\alpha_{\text{max}}R_{0}}).$$

Given estimates of *R*_0_ and *s*(0), we use equation (4.5) to obtain an estimate of this maximum serological attack rate $\left({\hat{\alpha }}_{\text{max}}\right)$, noting that this estimate is quite robust to a range of underlying spatial contact structures and variation in infective potential among individuals (Ma & Earn 2006).

In the second scenario, we assume that the eventual extinction of the epidemic is a result of the development of resistance in the wider community. The optimum attack rate is obtained by applying social distancing such that as the proportion of susceptibles in the community falls below 1/*R*_0_, the number of infectives is so small that the epidemic fades out without infecting a significant fraction of the remaining susceptible population. Here, the ultimate proportion of the population remaining susceptible is *s*(∞)=1/*R*_0_ (if *s*(∞)>1/*R*_0_, reintroduction of the infection could lead to another epidemic wave). This condition allows us to define *α*_min_=*s*(0)−1/*R*_0_ and, given estimates of *R*_0_ and *s*(0), we can obtain an estimate of this minimum attack rate $\left({\hat{\alpha }}_{\text{min}}\right)$. One would think that achieving this limit in practice should be rather difficult due to its extreme nature.

The difference between the scenarios arises due to the following reasons. Once the proportion of susceptibles in the community falls below 1/*R*_0_, the effective reproduction number drops below unity regardless of the degree of social distancing, and the epidemic is doomed to extinction. A freely flowing epidemic, however, overshoots *α*_min_ because at this stage the largest number of infectives is active. In the optimal case, social distancing is used to minimize the number of infectives at this stage, so that there is no overshoot. The ultimate attack rate therefore depends on how many individuals are infected as *R*(*t*) crosses 1.

In Sydney 1919, the attack rate must have lain between these extremes: $\alpha_{\text{min}}\leq \alpha \leq \alpha_{\text{max}}$. Based on the difference between our estimates of *α* and *α*_max_, we scale up the number of lives actually lost to estimate how many lives might have been lost if the epidemic had been entirely unimpeded by social distancing. By this measure, the number of deaths per 100 000 of the population that were prevented by social distancing (*Δ*) was $\Delta =(\alpha_{\text{max}}/\alpha -1)\pi \times 10^{5}$.

Whether or not social distancing has occurred during an epidemic, if it is relaxed (i.e. *σ*(∞)=1) during the final cases, it follows from equation (4.1) that

(4.6) $$R(\infty )=R_{0}(1-\alpha ).$$

Under optimal social distancing with a minimum possible attack rate $\left(\alpha =s(0)-1/R_{0}\right)$, we expect *R*(∞) to be unity. In epidemics where transmission is unimpeded (*σ*(*t*)=1 throughout), epidemic decline is much more rapid. During the final phase, there are sufficient infectious cases in that many susceptibles are infected, even though the reproduction number is well below 1.

### 4.5 Relationship between parameter estimates

To estimate the reproduction number during periods B and D ($\hat{R}(\text{B})$ and $\hat{R}(\text{D})$, respectively), we took the maximum of the smoothed estimate of *R*(*t*). Our estimate of the final reproduction number ($\hat{R}(\text{F})$) was the mean of the daily estimates during period F, weighted by the number of deaths on that day.

By substituting equation (4.4) into equation (4.1) and solving for *s*(0) after setting *σ*(B)=*σ*(F)=1, we obtain a relationship between *s*(0) and *α* along with the reproduction numbers during periods B and F and the associated cumulative number of *per capita* deaths,

(4.7) $$\hat{s}(0)=\frac{\alpha }{\pi (\hat{R}(\text{B})-\hat{R}(\text{F}))}\left\{\hat{R}(\text{B})\sum_{t^{\prime }=0}^{t_{\text{F}}+\tau }d(t^{\prime })-\hat{R}(\text{F})\sum_{t^{\prime }=0}^{t_{\text{B}}+\tau }d(t^{\prime })\right\}.$$

Here, *t*_B_ refers to the time until the peak in the effective reproduction number during period B, and *t*_F_ is the time to the middle of period F. We could have additionally used $\hat{R}(\text{D})$; however, *a priori* we were less confident that the population was behaving normally during period D.

All analyses were undertaken using the computing environment R v. 2.5.0 (R Development Core Team 2007).

## 5. Results and discussion

### 5.1 Estimating prior immunity, attack rate and degree of social distancing

Having estimated the values of $\hat{R}(\text{B})$ and $\hat{R}(\text{F})$, equation (4.7) establishes a unique relationship between *α* and *s*(0). The reported clinical attack rate is the obvious first choice as an estimate of *α*, but may be biased for several possible reasons: (i) it is conceivable that clinical cases may not have conferred solid immunity, (ii) cases that seroconvert may be asymptomatic, and (iii) illness may have been mistakenly attributed to influenza when it was in fact caused by another influenza-like illness (e.g. respiratory syncytial virus). Hence, we explore the values of *α* to be an arbitrary 10% below (0.329) and 10% above (0.403) the reported clinical attack rate. The upper value turns out to be just above the maximum possible under our final approach to estimating *α*; that is, to use equation (4.7) under the assumption that everyone was initially susceptible to infection (i.e. *s*(0)=1).

We therefore explore three estimates of *α*. For each estimate, we compute the corresponding values of *s*(0), *α*_max_, *α*_min_, *R*_A_(B), *R*_A_(D), *R*_A_(F) and *σ*_min_. We estimate *R*_0_ as the average of *R*_A_(B), *R*_A_(D) and *R*_A_(F).

Setting the serological attack rate to the observed clinical attack rate of 0.366 estimates the initial susceptible proportion to be *s*(0)=0.912 and ${\hat{R}}_{0}=1.76$ (table 2).

Setting the serological attack rate to *α*=32.9% (i.e. 10% lower than the clinical attack rate) corresponds to an initial susceptible proportion of *s*(0)=0.821. This scenario requires that 10% of those who developed clinical symptoms were not solidly protected against future severe attack, contradicting contemporary observations of influenza-dedicated hospital staff (Armstrong 1920).

If the population was initially fully susceptible (*s*(0)=1.0), a serological attack rate of *α*=0.401 is required to explain the epidemic dynamics. Again, if we assume that 0.366 is an accurate measure of the clinical attack rate, then it follows that 8.7% of those infected developed immunity without having developed clinical symptoms to the extent that they did not attend work. Although we do not give credence to a scenario that assumes *s*(0)=1.0, as it is probable that there was at least some heterotypic immunity from seasonal influenza, we note that it creates an upper bound of 8.7% for the fraction of infectives who could have been asymptomatic transmitters.

**Figure 4:**
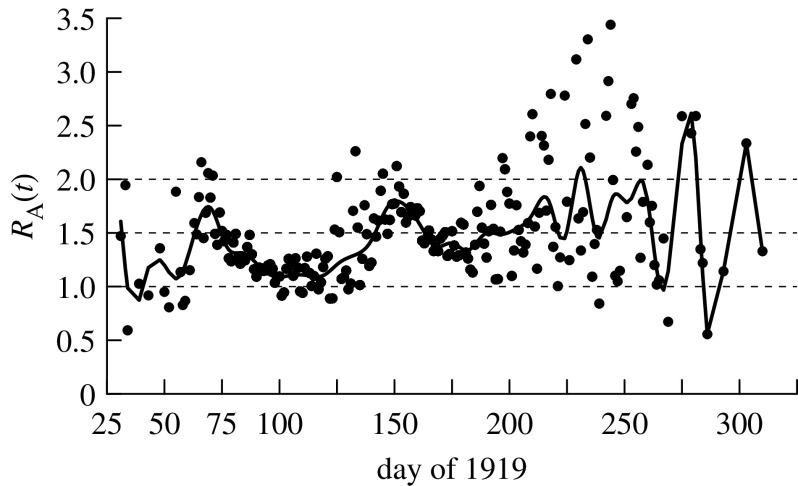
Reproduction number adjusted for the depletion of susceptibles, assuming a serological attack rate of 36.6 and 91.2% susceptibility at the start of the epidemic and the degree of social distancing. Fitted curve is a smoothed cubic spline curve. Day *t*=25 is 25 January 1919.

We suggest that, of these three estimates, the survey-based estimate of the clinical attack rate (0.366) is probably closest to the true value of the serological attack rate (i.e. *α*=0.366) and hence our preferred estimate of *R*_0_ is 1.76 (table 2; figure 4).

Each of the three scenarios returns the same value of *σ*_min_ (this is a mathematical consequence of our methods), corresponding to a reduction in the infectious contact rate of 38% during the first wave (table 2). During the second wave, the maximum estimated reduction in the infectious contact rate was less (24%). Interestingly, the second wave was perceived as being more severe than the first, so the difference between these values may be attributable to the public health policy of encouraging social distancing during the first wave. Alternatively, the difference could be explained by the exceptionally heavy rain that fell nearly throughout the month of May (following the first wave), thus discouraging people from getting out and circulating in the wider population (McCracken & Curson 2003).

Assuming homogeneous mixing, no social distancing, *s*(0)=31.2% and *R*_0_=1.76, using equation (4.5), we would expect an attack rate of 58.8%—much greater than the 36.6% observed. Assuming that the number of deaths is directly proportional to the attack rate, the reduction indicates that *Δ*=260 per 100 000 lives were possibly saved as a result of social distancing.

The estimated value of *α*_min_ was approximately 6% less than the modelled serological attack rate for the three parameter combinations examined. This suggests that few additional lives could have been saved by increasing the degree of social distancing, unless it was able to eliminate the epidemic. The observation that *R*(*t*) reduces to near 1 for a prolonged period during the last days of the epidemic further supports the conclusion that *α* was close to *α*_min_. Substituting *α*=0.588 into equation (4.6), the expected reproduction number during the final stages of the epidemic is 0.725—substantially less than the 0.95 observed.

**Figure 5:**
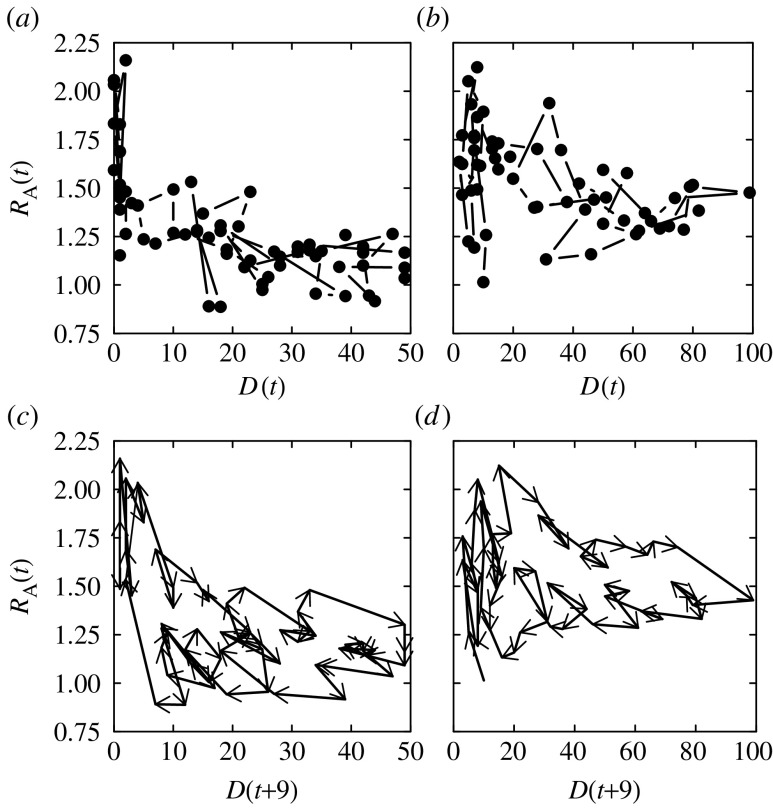
The relationship between the reproduction number adjusted for susceptible depletion (*R*_A_(*t*)) and the daily number of deaths (*D*(*t*)) during the (*a*) first and (*b*) second waves. The trajectory of the reproduction number based on deaths adjusted for the depletion of susceptibles in relation to the daily number of deaths advanced 9 days (*D*(*t*+9)) during the (*c*) first and (*d*) second waves.

The relationship between the adjusted reproduction number and the number of daily deaths for the first and second waves shows a negative trend—more deaths mean greater social distancing (figure 5*a*,*b*). For figure 5*c*,*d*, we wish to plot the reproduction number against the number of infections on the same day. Since the number of infections is unknown, we use the number of deaths 9 days later as a proxy. The clockwise cycles reveal the delay between the infection and the subsequent decline in *R*_A_(*t*)—and hence the degree of social distancing.

We have assumed an ‘all or nothing’ model of prior immunity, meaning that a fraction of individuals were totally protected from infection during the pandemic period. The main alternative model of prior immunity is that a fraction of the population is partially immune, having a lower (but non-zero) risk of infection. Under some circumstances, there will be material differences between the behaviour of these prior immunity models: if *R*_0_ is very large, all susceptibles, whether fully or partially immune, will inevitably be infected; alternatively, if there is assortative mixing between classes of susceptibles, fully susceptibles will be overrepresented during the early stages of the epidemic and underrepresented in later stages. These circumstances do not apply to the Sydney 1919 epidemic—there was a reasonably low attack rate (less than 50%) and little evidence to support strongly assortative mixing. While our model result is that 10% of the population were fully immune, for these data we cannot easily distinguish this from alternatives, such as where 20% of the population had 50% of the normal risk of infection.

While the infectivity profile we use has empirical support, it is interesting to consider the effect of changing the infectivity profile. Had we used an infectivity profile with a shorter mean serial interval, we would have obtained reproduction numbers closer to 1, meaning that smaller changes in the degree of social distancing would explain the epidemic waves. However, the reproduction number cannot be reduced much below 1.6 before it becomes impossible to achieve an attack rate of 36.6%, in an epidemic with two waves of similar magnitude. On the other hand, a longer serial interval would have produced higher estimates of *R*_0_. In this case, we have underestimated the social distancing achieved during the 1919 epidemic.

It is possible that other interventions, such as closing schools and quarantining infectives, played a role in containing the epidemic. We argue that most of these can be broadly categorized as social distancing. Measures such as quarantine are likely to have been practised more or less constantly throughout the epidemic and probably did not contribute to the changes in *R*(*t*).

## 6. Conclusions

We conclude that the variable application of social distancing, whereby individuals reduced their infectious contact rate in response to the perceived risk, is a plausible explanation for the multiple waves of pandemic strain influenza seen during 1919 in Sydney, Australia. Indeed, while the waxing and waning of the multiple waves appears dramatic, the degree of social distancing required to explain this (in this case, at most, halving one's infectious contact rate) seems quite possible. More generally, Bootsma & Ferguson (2007) and Hatchett *et al*. (2007) have demonstrated that variation in the timing of introduction and lifting of non-pharmaceutical interventions aimed at reducing contact rates can explain why cities experienced different inter-wave periods, ranging from being so short as to be undetectable through to several months (Taubenberger & Morens 2006). We note, however, that transient social distancing certainly does not explain why the case-fatality rate of the 1918–1919 pandemic typically was higher during the second wave, as indeed was the case for Sydney (McCracken & Curson 2003). However, note that the very similar reproduction numbers observed during both waves of the epidemic support our initial assumption that *R*_0_ did not differ over the course of the epidemic.

Subject to the assumption that infection at any time conferred protection against a subsequent severe attack, we conclude that approximately 9% of the population were resistant to the epidemic strain prior to the epidemic, and that, during the epidemic, not more than approximately 9% of infections that conferred resistance to the epidemic strain were subclinical to the extent that people were able to continue working. Using our best estimate that 91.2% of individuals were initially susceptible, the *R*_0_ of the 1919 influenza epidemic in Sydney was 1.8, consistent with recent estimates that have used a similar mean serial interval (Ferguson *et al*. 2005; Sertsou *et al*. 2006). The observed attack rate, however, was substantially less than would be expected for this basic reproduction number, and we argue that social distancing is a plausible reason for this. This result underlines the effective role that social distancing could possibly play in mitigating the effects of a future pandemic of influenza.

## Figures and Tables

**Table 1:** Summary of epidemic incidence, policy and individual's perceived risk factors influencing the degree of social distancing (*σ*(*t*)) during different periods of the influenza epidemic in Sydney 1919. (Day 1 is 1 January 1919. The question mark assigned to *σ*(*t*) during period D reflects our uncertainty surrounding whether people fully resumed normal contact behaviour at some stage during this period.)

period	day, 1919	incidence	drivers of perceptions	perceived risk	*σ*(*t*)
A	32–59	very low	high publicity and policy	high waning	<1
B	60–84	low	threat evidently passed	low	1
C	85–134	high	high incidence and policy	high	<1
D	135–165	moderate	decreased incidence	moderate	1(?)
E	166–223	high	high incidence	high	<1
F	224 onwards	low	threat passed	low	1

**Table 2:** Values of the attack rate *α* and the corresponding values of the initial susceptible proportion (*s*(0)), the basic reproduction number (*R*_0_), the minimum and maximum fractions that could have been infected (*α*_min_, *α*_max_), the adjusted reproduction numbers during periods when we expect that social distancing is at a minimum (*R*_A_(B), *R*_A_(D) and *R*_A_(F)), the social distancing coefficient when social distancing was at its greatest (*σ*_min_), and the estimated number of deaths avoided per 100 000 (*Δ*). (Values in the first row are computed by assuming that *α* was 10% less than the reported clinical attack rate with *s*(0) allowed to vary freely. The second row is computed using the clinical attack rate as an estimator for *α*. The third row is computed by adjusting *α* to obtain *s*(0)=1.0.)

*α*	*s*(0)	*R*_0_	*α*_min_	*α*_max_	*R*_A_(B)	*R*_A_(D)	*R*_A_(F)	*σ*_min_	*Δ*
0.329	0.821	1.96	0.309	0.529	1.94	1.97	1.93	0.619	260
0.366	0.912	1.76	0.344	0.588	1.75	1.80	1.74	0.619	260
0.402	1.000	1.60	0.377	0.644	1.59	1.64	1.58	0.619	260
